# A Bidirectional Knudsen Pump with a 3D-Printed Thermal Management Platform

**DOI:** 10.3390/mi12010058

**Published:** 2021-01-06

**Authors:** Qisen Cheng, Yutao Qin, Yogesh B. Gianchandani

**Affiliations:** 1 Center for Wireless Integrated MicroSensing and Systems (WIMS2), University of Michigan, Ann Arbor, MI 48109, USA; 2 Department of Mechanical Engineering, University of Michigan, Ann Arbor, MI 48109, USA; 3 Department of Electrical Engineering and Computer Science, University of Michigan, Ann Arbor, MI 48109, USA

**Keywords:** gas chromatography, gas flow, nanoporous membranes, microactuator

## Abstract

This paper reports on a bidirectional Knudsen pump (KP) with a 3D-printed thermal management platform; the pump is intended principally for microscale gas chromatography applications. Knudsen pumps utilize thermal transpiration, where non-viscous flow is created against a temperature gradient; no moving parts are necessary. Here, a specialized design leverages 3D direct metal laser sintering and provides thermal management that minimizes loss from a joule heater located on the outlet side of KP, while maintaining convective cooling on the inlet side. The 3D-KP design is integrative and compact, and is specifically intended to simplify assembly. The 3D-KP pumping area is ≈1.1 cm^2^; with the integrated heat sink, the structure has a footprint of 64.2 × 64.2 mm^2^. Using mixed cellulose ester (MCE) membranes with a 25 nm average pore diameter and 525 μm total membrane thickness as the pumping media, the 3D-KP achieves a maximum flow rate of 0.39 sccm and blocking pressure of 818.2 Pa at 2 W input power. The operating temperature is 72.2 °C at ambient room temperature. In addition to MCE membranes, anodic aluminum oxide (AAO) membranes are evaluated as the pumping media; these AAO membranes can accommodate higher operating temperatures than MCE membranes. The 3D-KP with AAO membranes with 0.2 μm average pore diameter and 531 μm total membrane thickness achieves a maximum flow rate of 0.75 sccm and blocking pressure of 496.1 Pa at 9.8 W at an operating temperature of 191.2 °C.

## 1. Introduction

Bidirectional gas flow is necessary in a number of application contexts. Certain analytic instruments such as gas chromatographs [1,2,3,4,5] and mass spectrometers [6,7] require gas flow in opposing directions for sampling and analysis steps. In microfluidic systems, bidirectional pumping is often employed to support pneumatic operations or valve control for sample transport and processing [8,9,10]. It has also been used to eliminate the need for valves [11,12].

Knudsen pumps (KPs) have been shown to provide bidirectional pumping without moving parts [11,12,13]. KPs utilize the principle of thermal transpiration, wherein the net flux of gas molecules is induced against the temperature gradient in the direction from the low temperature region to the high temperature region [14]. This flow is evident only when viscous flow is suppressed, i.e., under conditions where the collisions between gas molecules do not greatly dominate collisions between gas molecules and the walls of the flow channel. Operation at atmospheric pressure is possible even with hydraulic diameters of ≈2 μm [15]. The pumping direction can be reversed by powering different heaters located at opposing ends of the flow channel. In the absence of moving parts, KPs eliminate friction and stiction issues that affect other types of micropumps, operating quietly and avoiding wear in motion. The motionless nature of KPs also results in a relatively simple architecture, which can be easily scaled and integrated in different microsystems.

Two performance indicators for KPs are the flow rate and the blocking pressure. A full theoretical analysis usually requires Monte Carlo simulations, which become intractable for complex geometries. Sharipov’s equation [16] is an approximation of the Boltzmann equation that can be used to estimate the mass flow rate of thermal transpiration through a circular narrow channel:

(1) $$M=\left(\frac{Q_{T}\Delta T}{T_{avg}}-\frac{Q_{P}\Delta P}{P_{avg}}\right)\frac{\pi a^{3}P_{avg}}{l}{\left(\frac{m}{2k_{B}T_{avg}}\right)}^{0.5}$$

where *M* is the mass flow rate; *T_avg_* and Δ*T* are the average temperature and temperature difference between the hot and the cold end of the channel, respectively; *P_avg_* and Δ*P* are the average pressure and pressure head (i.e., pressure difference) between two ends of the channel, respectively; *a* and *l* are the diameter and length of the channel, respectively; *m* is the gas molecular mass; *k_B_* is the Boltzmann constant; and *Q_T_* and *Q_P_* are the coefficients of thermally-driven flow and pressure-driven flow, respectively. The values of *Q_T_* and *Q_P_* have been tabulated by Sharipov [16]. The maximum pressure head, i.e., the blocking pressure (Δ*P_eq_*), is reached when the flow rate is reduced to zero and can be derived from Equation (1) as:

(2) $$\Delta P_{eq}=\frac{Q_{T}}{Q_{P}}\frac{P_{avg}}{T_{avg}}\Delta T$$

For a given pumping medium and structure operating at a given pressure, a higher Δ*T* results in a higher flow rate or blocking pressure. The coefficients *Q_T_* and *Q_P_* are mostly determined by the pumping channel geometry, which is constrained by manufacturing or material selection. The parameter that is under the control of the device designer is Δ*T*. In typical unidirectional implementations of KP that are intended to operate at atmospheric pressure, arrays of nanoscale channels can be provided by either micromachining silicon [15,17,18,19] or by nanoporous media such as ceramics, zeolites, and polymer membranes [20,21,22,23]. The nanoscale channels are sandwiched between a passive heat sink and a Joule heater to establish the temperature gradient that generates thermal transpiration flow.

The measured performance of silicon micromachined KPs [15,18,19] can be within 15% of the estimate provided by Sharipov’s equation. Pressure-driven viscous flow is usually more significant in the KPs that use nanoporous materials because of the nature of these media (which have more tortuosity, non-uniformity, and random defects). Such viscous flow is in the direction opposite to thermal transpiration flow, and thus causes deviation from the equations. Some prior works introduced correction factors (e.g., a leakage aperture diameter) to reduce the difference between measurements and predictions [20,21,22,23].

For bidirectional pumping, it is necessary to provide a way to reverse the temperature gradient across the pumping media. An early example of a bidirectional KP incorporated thermoelectric elements into the KP architecture [13], allowing reversal of the pumping by reversing the direction of the electrical current. In later work, bidirectional KPs were implemented by directly attaching heat sinks and Joule heaters on both sides of the nanoporous membranes [11,12,24]. Despite past success, effective implementation of both cooling and heating functions on each side of a bidirectional KP remains an open challenge. The heating and cooling functions generally require contradictory thermal management depending upon the pumping direction. In particular, efficient heat dissipation contributes to a large Δ*T* when it is implemented at the cool upstream side, but significantly decreases the Δ*T* when the flow is reversed. To address these problems, a more deliberate design is needed for the bidirectional KP architecture.

In this paper, we present a 3D-printed bidirectional KP (3D-KP) that addresses these problems. In contrast with prior work, the architecture of the 3D-KP is customized to provide superior thermal management in order to generate an appropriate Δ*T* for pumping in both directions. By design, this architecture exploits direct laser metal sintering (DMLS), which is one of the latest 3D metal printing technologies [25]. The architecture is also integrative and compact, permitting a very simple assembly step. Using this 3D-printed architecture, stacks of both mixed cellulose ester (MCE) membranes and anodic aluminum oxide membranes are evaluated as the pumping medium. Compared to mixed cellulose ester (MCE) membranes, which have a maximum operating temperature of only ≈75 °C, the AAO membranes can endure a much higher temperature of 400 °C [26]. The design and modeling results are described in Section 2, the fabrication and assembly are described in Section 3, the experimental results are described in Section 4, and the discussion and conclusions are described in Section 5.

## 2. Design and Modeling

### 2.1. Architecture

**Figure 1:**
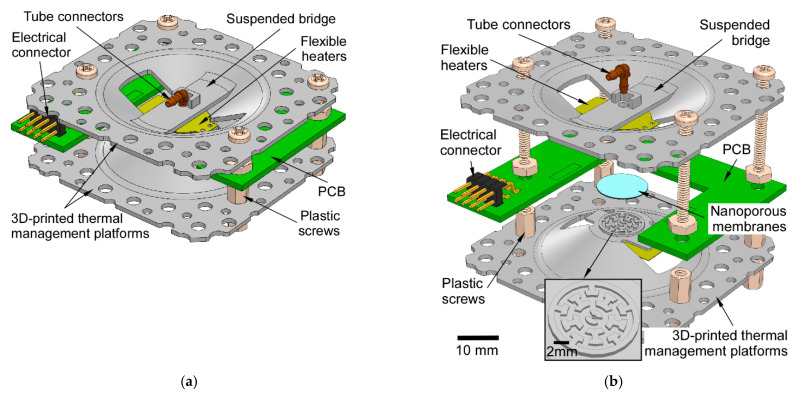
A 3D model of the 3D-printed bidirectional Knudsen pump: (**a**) full assembly; (**b**) exploded view. The architecture sandwiches the pumping element (a stack of nanoporous membranes) between two identical 3D-printed thermal management platforms.

The 3D-printed bidirectional Knudsen pump (3D-KP) has a simple architecture that features two 3D-printed thermal management platforms (Figure 1). The thermal management platforms provide gas flow routing, heat dissipation, and mechanical support and protection for the other components.

The design was reached iteratively with major considerations of heat transfer efficiency, thermal mass, and manufacturing constraints. The final design ensured both effective conductive heating on the hot side and convective cooling on the cool side. The overall size of the thermal management platform was tailored to balance sufficient heat dissipation surface area and minimize thermal mass. Consequently, both the static and transient performance were considered. Some dimension choices (i.e., thickness, perforation sizes) were constrained by either structural robustness or manufacturing restrictions. Details can be found in Section 2.2 and Section 3.1.

**Figure 2:**
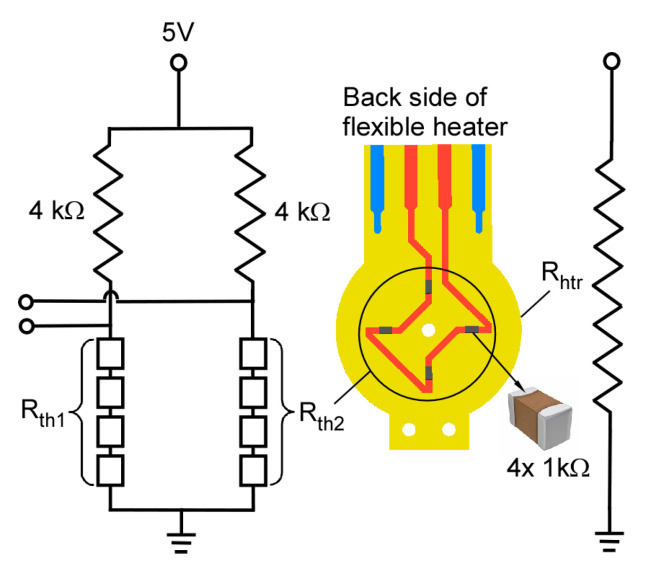
Design of the customized flexible heater with heating metal trace (*R*_htr_) on the front side and integrated thermistors (*R*_th1_, *R*_th2_) on the back side. An external voltage divider circuit is used to measure the change of electrical resistance of the thermistors.

A stack of nanoporous membranes serving as the pumping element was sandwiched between the two thermal management platforms. In this work, we used a stack of 5 mixed cellulose ester (MCE) membranes (Millipore, Burlington, MA, USA) and a stack of 9 anodic aluminum oxide (AAO) membranes (Sigma-Aldrich, St. Louis, MO, USA) as two different thermal transpiration media. The MCE membrane had a pore size of 25 nm (Knudsen number, Kn ≈ 2.7), a porosity of 70%, and a thickness of 105 μm. The AAO membrane had a pore size of 0.2 μm (Kn ≈ 0.3), a porosity of 25–30%, and a thickness of 59 μm. Both membranes were circular in shape with a diameter of 12 mm (providing an effective pumping area ≈ 1.1 cm^2^). A customized flexible heater (Figure 2) with integrated resistive thermistors (NCP18XH102, Murata Electronics, Smyrna, GA, USA) was mounted on the center of each thermal management platform to provide heat. A printed circuit board (PCB) was positioned between the two thermal management platforms to provide electrical connection between the flexible heaters and external circuitry. Two standard tube connectors were anchored on the suspended bridges of thermal management platforms to interface with external tubing. The thermal management platforms and the PCB were screwed together to form the 3D-KP architecture.

**Figure 3:**
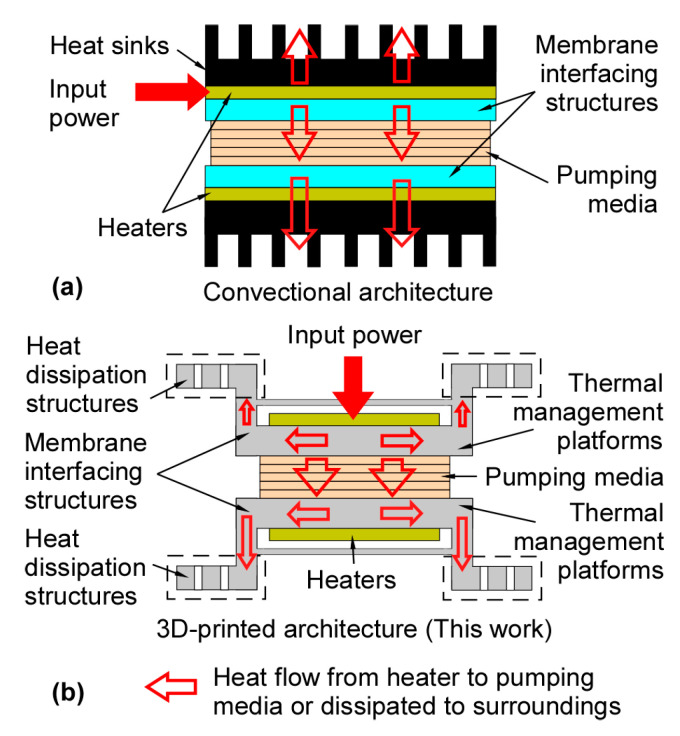
Comparison between previously reported KPs and the 3D-KP architecture. (**a**) The conventional architecture [11,12,24], where one surface of each heater is in full contact with a heat sink. (**b**) The 3D-KP architecture, which arranges the heaters further away from the heat dissipation structures. This reduces the waste of heat through the heat dissipation structure on the hot side, while not impeding heat dissipation on the cool side.

The 3D-KP architecture was designed to have superior thermal performance over prior work (Figure 3). In previously reported bidirectional KPs [11,12,24] (Figure 3a), the arrangement of elements on each side of the membrane included a membrane interfacing structure (implemented as glass dies with micromachined fluidic channels), a joule heater, and a heat sink. In this arrangement, one surface of each heater was in full contact with a heat sink, which resulted in a large fraction of heat being wasted to the surroundings through the heat sink rather than being used for pumping. In the 3D-KP architecture (Figure 3b), the membrane interfacing structure and a heat dissipation structure (which serves the purpose of a heat sink) were integrated into a monolithic thermal management platform. The thermal management platform was designed in a way that accommodates a heater next to its membrane interfacing structure and further away from its heat dissipation structure. Consequently, the waste of heat through the heat sink was reduced on the hot side, while the heat dissipation reminded effective on the cool side. This is discussed further in Section 2.3.

### 2.2. 3D-Printed Components

**Figure 4:**
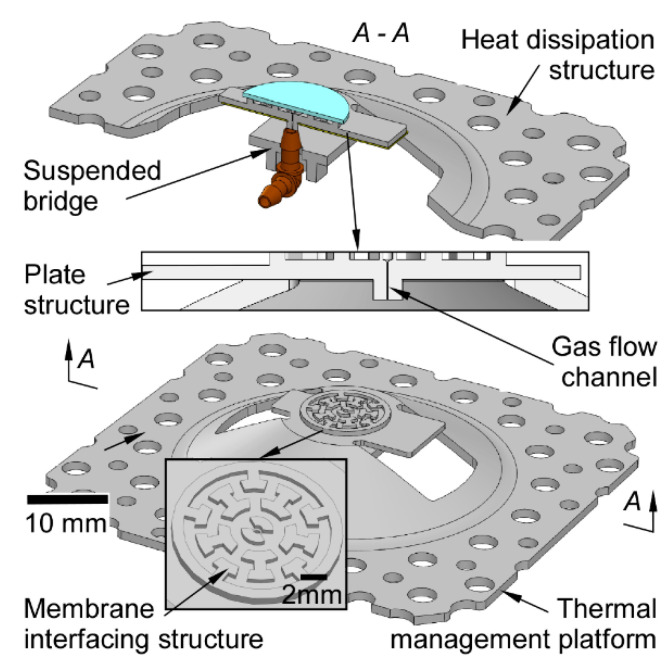
The model of the 3D-printed thermal management platform. Each thermal management platform consists of a heat dissipation structure, a plate structure to interface with the heater, a membrane interfacing structure, a suspended bridge, and a gas flow channel to route the flow from the membranes to the outside.

The 3D-KP architecture depends on the customized thermal management platforms, which are built using 3D printing technology (Figure 4). Each thermal management platform is composed of a gas flow channel, a membrane interfacing structure, a plate structure, a suspended bridge, and a heat dissipation structure. Gas flow is routed across the nanoporous membranes and then through the embedded gas flow channel and the tube connector. The gas flow channel is 0.4 mm in diameter and 1 mm in length. The nanoporous membranes are in contact with the membrane interfacing structure. The membrane interfacing structure is designed to both allow effective heat conduction between the thermal management platform and the membranes, and direct gas flow from the membranes to the gas flow channel. The diameter of the effective pumping area (≈11.8 mm) is determined by the membrane interfacing structure of the thermal management platform. The flexible heaters are attached to the plate structure. The plate structure and the membrane interfacing structure are designed to be thin enough so that the distance between the heater and nanoporous membranes is minimal, in this case 1.4 mm. The suspended bridge enhances the structural strength of the platform, which prevents collapse in the 3D printing process. A tube connector is also positioned and anchored on the suspended bridge. The heat dissipation structure serving as a heat sink is 1.2 mm thick. It incorporates a large number of perforations to increase the thermal convection surface area for more efficient natural convective cooling. The perforations include 16 through-holes of 1.5 mm diameter and 26 through-holes of 2.5 mm diameter.

### 2.3. Thermal Performance Modeling

The transient thermal performance of the 3D-KP was simulated using finite element analysis (FEA) in COMSOL 5.3. The simulation had two purposes. First, it verified that sufficient heat flow was directed across the pumping medium instead of being directed into the dissipation structure of the thermal management platform. Second, it estimated the maximum temperature difference (Δ*T*) across the membrane stack; this determines the pump performance as indicated in Equations (1) and (2). The maximum Δ*T* is additionally constrained by the maximum operating temperature of the nanoporous membranes. Because AAO membranes can endure much higher temperature (≈400 °C) than the MCE membranes (≈75 °C), the model was built to evaluate the limit of MCE membranes. The major simulated parameters are listed in Table 1.

**Figure 5:**
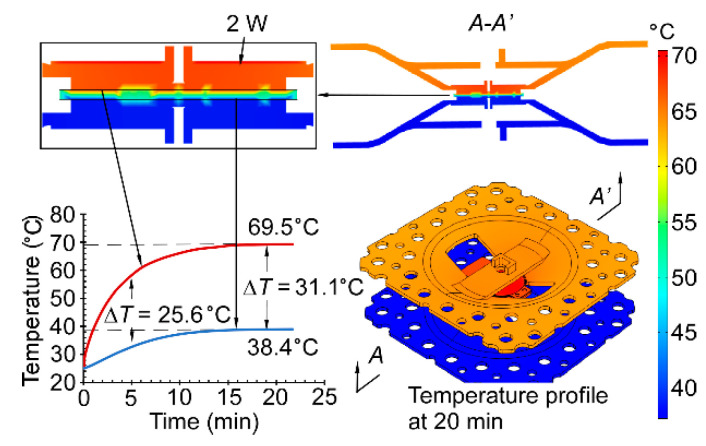
Transient finite element analysis (FEA) of the 3D-KP thermal response in COMSOL. The temperature profiles show that heat is concentrated within the pumping area of the KP. At 20 min, the largest temperature difference (Δ*T*) between the hot and cool sides of the MCE membranes is ≈31.1 °C, with a power of 2 W.

The FEA results are shown in Figure 5. The temperature profiles indicate that the heating is mostly concentrated within the plate structure of the thermal management platform on the hot side. This design eliminates the direct short thermal path that exists between the heater and the platform in the conventional KP structures. A fraction of heat is dissipated to the ambient through natural convection. Simulations showed that the Δ*T* between the hot and cool sides of the MCE membranes was ≈31.1 °C at 2 W. This was much larger than the Δ*T* (= 23.2 °C) measured for a prior conventional KP architecture using the same MCE membranes [24]. The temperature on the hot side of the MCE membranes was about 69.5 °C at 2 W, which was below the maximum operating temperature.

## 3. Fabrication

### 3.1. 3D Printed Metal

**Figure 6:**
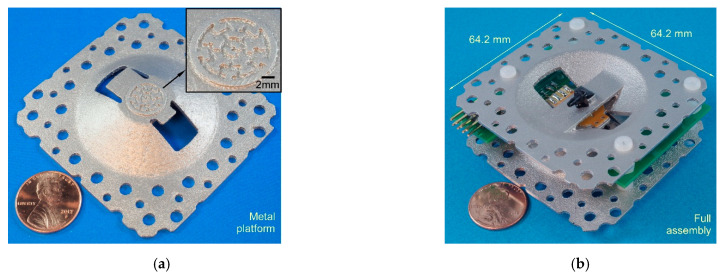
Photos of a 3D-printed bidirectional KP: (**a**) 3D-printed thermal management platform using DMLS process; (**b**) fully assembled KP.

The thermal management platforms (Figure 6a) in the 3D-KP were 3D-printed using the direct metal laser sintering (DMLS) process. This process uses high-energy lasers to fuse metal powders layer-by-layer, constructing 3D structures digitally. The finest resolution of the commercial DMLS process is about 20 μm. Generally, at least 20 layers are required to successfully build a structural feature. Therefore, the minimum feature size in the design was selected to be 0.4 mm. To eliminate continuous pores that would allow air to leak through structural features, the minimum thickness of the structural features in the thermal management platform was designed to be 1 mm. The specifications of the DMLS service (Star Rapid Manufacturing Ltd., Guangdong, China) are listed in Table 2.

Aluminum alloy (AlSi_10_Mg) was selected to build the thermal management platform using the DMLS process. This alloy has relatively high thermal conductivity and low thermal mass among the 3D printable metal materials [27]. These properties are preferable for bidirectional KPs, which require both efficient heating and cooling.

### 3.2. Assembly Process

Two 3D-KPs were assembled using either MCE membranes and AAO membranes. The nanoporous membranes were sandwiched between the two thermal management platforms. Five MCE membranes were stacked together and assembled with a pair of thermal management platforms. A viscous epoxy (Stycast2850FT, Henkel, Germany) was used to seal the edges of the membranes. A stack of AAO membranes was also assembled with another pair of thermal management platforms in a similar approach. To match the total thickness of the two membrane stacks, 9 AAO membranes were used in the assembly. The flexible heaters were placed on the thermal management platform with an adhesive film on the flexible heater. The tube connectors were anchored to the thermal management platform with epoxy.

The components were finally assembled together using plastic screws and nuts. Plastic screws and nuts have low thermal conductivity, so the thermal cross-talk between the hot and cool sides was reduced. The full assembly had a footprint of 64.2 × 64.2 mm^2^.

## 4. Experimental Results

### 4.1. Test Setup

**Figure 7:**
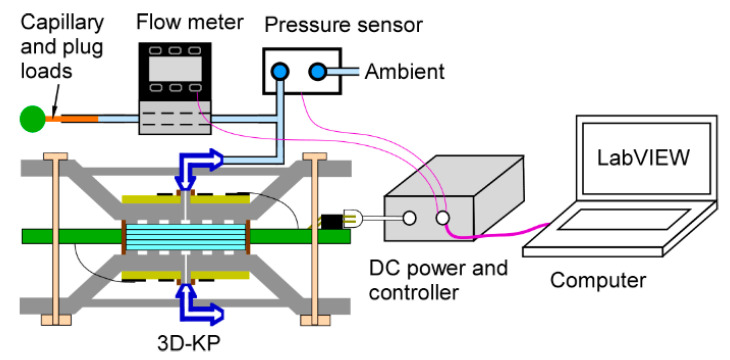
Testing setup for 3D-KPs. A flow meter is connected downstream of the KP and a pressure sensor is connected in parallel with the KP. Flow loading is adjusted using a capillary tube and a rubber plug. The pumping direction and power levels are controlled by the LabVIEW program.

Both 3D-KPs, one with MCE membranes (3D-KP.MCE) and the other with AAO membranes (3D-KP.AAO), were tested using the same test setup and methods to measure the flow rate characteristics at various pressure heads (Figure 7). A commercial flow meter (MW-20SCCM-D, Alicat Scientific, Inc., Tuscon, AZ, USA) with negligible flow resistance was connected downstream of the 3D-KP to continuously measure the volume flow rate pumped by the 3D-KP. A commercial differential pressure sensor (MPX5010DP, NXP Semiconductor Inc., Austin, TX, USA) was connected in parallel with the 3D-KP to continuously measure the pressure head between the inlet and the outlet of the pump. The 3D-KPs were tested under three conditions: the minimally loaded or fully open condition; a partially loaded condition, in which a capillary tube (with inner diameter ≈ 100 µm, length ≈ 10 cm) was incorporated into the flow path; and the maximally loaded or fully blocked condition, in which the flow was stopped by a rubber plug. Both 3D-KPs were tested in the air with a relative humidity of 31%.

The maximum power used for each 3D-KP was mainly limited by the maximum operating temperature of the nanoporous membranes. The 3D-KP.MCE pump was tested with power levels of 1, 1.5, and 2 W. The 3D-KP.AAO pump was tested from 2 to 9.8 W. Both the forward and reverse pumping performance were tested for each 3D-KP. The forward pumping was established by powering the flexible heater on the lower side of the 3D-KP. A custom LabVIEW program controlled the direction, start, and end of pumping. The flow rate, pressure head, and temperature readouts were also continuously recorded by the program.

### 4.2. Steady-State Performance

**Figure 8:**
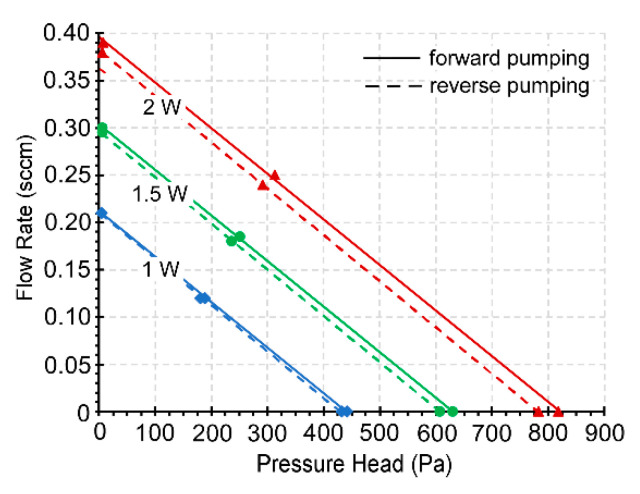
Steady-state performance of the 3D-KP.MCE showing the flow rate against the pressure head at three power levels. The forward and reverse pumping are almost identical.

**Figure 9:**
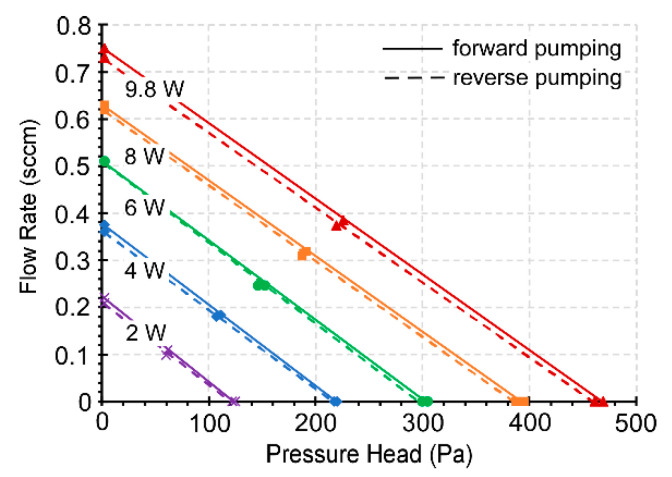
Steady-state performance of the 3D-KP.AAO showing the flow rate against the pressure head at five power levels. The AAO membranes can tolerate higher operating power than MCE membranes. The forward and reverse pumping are almost identical.

With 2 W power applied to the 3D-KP.MCE, the pump provided a maximum air flow rate of 0.39 sccm (≈0.36 sccm/cm^2^) in the fully open condition for forward pumping and 0.38 sccm (≈0.35 sccm/cm^2^) for reverse pumping (Figure 8). In the fully blocked condition, the blocking pressures measured were 818.2 and 782.4 Pa for forward and reverse pumping, respectively. With the same power of 2 W applied to the 3D-KP.AAO, the performance was much weaker compared to 3D-KP.MCE. However, at 9.8 W, the 3D-KP.AAO achieved a maximum flow rate of 0.75 sccm (≈0.69 sccm/cm^2^) for forward pumping and 0.73 sccm (≈0.67 sccm/cm^2^) for reverse pumping (Figure 9). This was nearly 2× the maximum flow rate available from 3D-KP.MCE at 2 W. The maximum blocking pressures for the 3D-KP.AAO were measured as 469.1 and 462.5 Pa for forward and reverse pumping at 9.8 W, respectively.

The performance of both 3D-KPs increased proportionally with the input power. The relationship between the flow rate and the applied pressure was approximately linear. In general, the performance was almost symmetrical between forward and reverse pumping. The slight difference of performance between the forward and reverse pumping was mainly caused by the thermal asymmetry between the upper and lower thermal management platforms, which is addressed in Section 5.

### 4.3. Transient Performance

**Figure 10:**
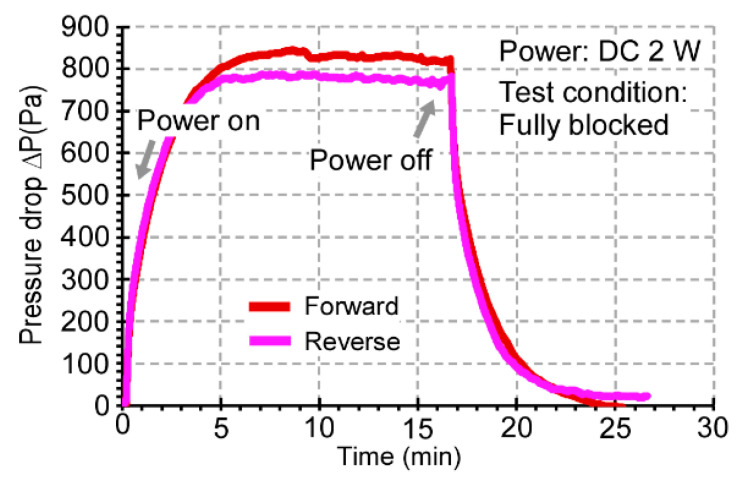
Transient profile of blocking pressure between the two flow ports of the KP with MCE membranes in forward and reverse pumping at 2 W applied power. The performance was stabilized in ≈5 min in both cases.

The transient performance of the 3D-KP was tested by measuring the blocking pressure over time. With 2 W applied to the 3D-KP.MCE, the pressure stabilized after 5 min for both forward and reverse pumping (Figure 10). This showed that the response times for 3D-KP to reach the steady-state performance were about 5 min. The measured response times matched the predicted response time of the temperature difference Δ*T* across the MCE membrane stack in the FEA simulation. The 3D-KP.AAO response times were similar for both forward and reverse pumping. Because the volume of the inner reservoir was negligible, the response time was dominated by the thermal time constant.

### 4.4. Operating Temperature Measurements

The operating temperatures of both pumps were measured with commercial thermistors located on the back side of their embedded flexible heaters (Figure 2). Electrical resistance values of the thermistors gradually decreased from the nominal value of 1 kΩ with rising temperature. The temperatures can be calculated from the measured resistance with the *β*-parameter Equation [28]:

(3) $$\frac{1}{T}=\frac{1}{T_{0}}+\frac{1}{\beta }\ln\left(\frac{R}{R_{0}}\right)$$

where *T* is the temperature to be calculated; *T*_0_ is the initial temperature, which is usually the room temperature (≈25 °C); *R* is the measured resistance; *R*_0_ is the initial resistance, which is usually the nominal resistance; *β* is a constant parameter determined by the type of thermistor. The thermistors used in this work were calibrated to have *β* around 4100.

Selected measurements of the operating temperature for 3D-KP.MCE and 3D-KP.AAO are listed in Table 3 for comparison. At 2 W, the 3D-KP.MCE achieved a maximum temperature difference (Δ*T*) of 39.6 °C for forward pumping and 36.5 °C for reverse pumping. The 3D-KP.AAO achieved only about 56% of the 3D-KP.MCE at the same input power of 2 W. Considering that the total thickness of the two membrane stacks was the same, the temperature measurements indicate that the effective thermal conductance of AAO membranes was approximately twice that of the MCE membranes. The highest Δ*T* of 3D-KP.AAO was ≈121.2 °C for forward pumping and ≈118 °C for reverse pumping with an input power of 9.8 W. This outcome indicates that the AAO membranes may be used over a wider range of temperatures than MCE membranes. In the tests of 3D-KP.AAO, the highest temperature on the hot side was maintained below 200 °C to avoid damage to other components (i.e., plastic tube connectors, screws, and nuts).

The temperatures observed on the cool side (*T_cool_*) were close to the temperatures predicted in FEA simulations. The temperatures on the hot side (*T_hot_*) were the major contributions to the discrepancy between the measured and predicted Δ*T*. Even so, the overall difference between the measurements and the simulations of Δ*T* was <18%.

**Figure 11:**
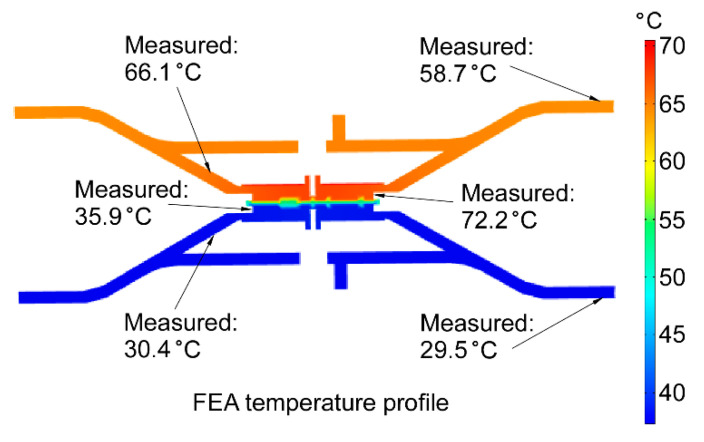
Comparison between measured temperature distribution and predicted profile from the FEA. At 2 W, the measured Δ*T* across nanoporous membranes was ≈36.3 °C, which was ≈14.3% higher than predicted.

The temperature distribution along the thermal management platforms (Figure 11) was measured using a commercial thermocouple. In the 3D-KP.MCE, at 2 W, the highest temperature of the thermal management platform on the hot side was measured at the membrane interfacing structure (≈72.2 °C), which was in contact with the flexible heaters. The temperatures along the heat dissipation structure of the thermal management platform on the hot side were ≈66.1 °C near the center and ≈58.7 °C at the edge. The temperatures of the thermal management platform on the cool side were ≈35.9 °C at the membrane interfacing structure, ≈30.4 °C near the center, and ≈29.5 °C at the edge of heat dissipation structure. The measured distribution matched the predicted profile in the FEA, with minor discrepancies. The temperature distribution indicated that the heating was concentrated within the pumping area and the heat dissipation structure on the cool side was effective.

### 4.5. Chromatography Tests

**Figure 12:**
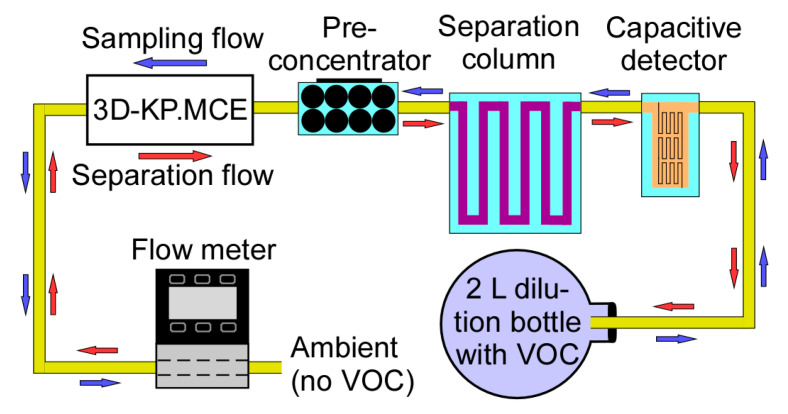
A micro gas chromatography system, including a preconcentrator, a separation column, a capacitive detector, and the 3D-KP.MCE. The test vapor was prepared in a 2 L dilution bottle. An external flow meter was connected to monitor the flow rate.

In order to assess the practical effectiveness of the 3D-KP architecture, the 3D-KP.MCE was used to support a micro gas chromatography system, which consisted of a preconcentrator, a separation column, and a capacitive detector. These are microfabricated components reported in our prior work [12]. The preconcentrator contained Carbopack-B and Carbopack-X absorbents (Sigma Aldrich, St. Louis, MO, USA) to trap the volatile organic compounds (VOCs) at room temperature during sampling. The preconcentrator also incorporated a thin-film metal heater and thermistor to provide thermal desorption during separation. The separation column was a 30-cm-long channel with a hydraulic dimeter of 230 μm and coated with a 0.2-μm-thick OV-1 (Ohio Valley Specialty, OH, USA) stationary phase for separation of VOCs. The capacitive detector was a thin-film metal interdigitated capacitor coated with 0.3-μm-thick OV-1. The pump, preconcentrator, separation column, and detector were connected in series (Figure 12). In order to measure the flow rate during operation, a flow meter (MW-20SCCM-D, Alicat Scientific, Inc., Tuscon, AZ, USA) was connected to the flow path. A VOC mixture sample was prepared in a 2 L dilution bottle, which contained 6 compounds at equal concentrations of 1 p.p.m, including acetone, benzene, toluene, ethylbenzene, o-xylene, and mesitylene.

**Figure 13:**
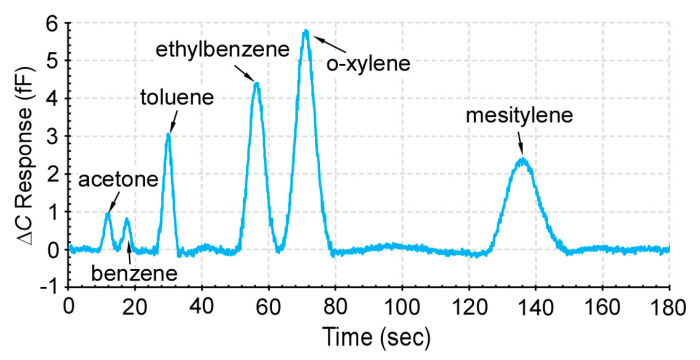
A typical chromatogram provided by the gas chromatography system, which was supported by the 3D-KP.MCE. Six different chemicals were successfully collected, separated, and detected.

During the test, the 3D-KP.MCE was first operated in reverse pumping mode at 3 W to provide a flow rate of 0.26 sccm in order to sample the VOCs from the dilution bottle into the preconcentrator. After 40 min of sampling, the 3D-KP.MCE was turned off for ≈4 min to cool down. The 3D-KP.MCE was then operated in forward pumping mode at 3 W to provide a flow rate of 0.3 sccm. After 20 s pumping, the preconcentrator was heated to 200 °C for 10 s to thermally inject the VOCs. The separation column and detector were operated at room temperature (22 °C). As evident from the resulting chromatogram (Figure 13), the 6 tested VOCs were well separated and detected.

## 5. Discussion and Conclusions

**Figure 14:**
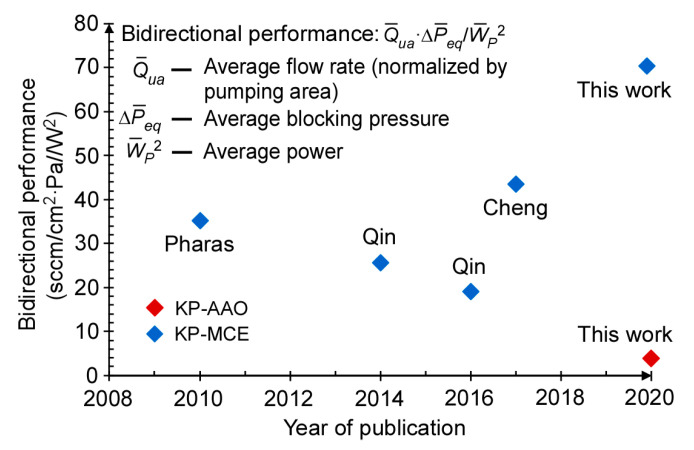
Comparison of bidirectional performance among prior and 3D-printed bidirectional KPs. The bidirectional performance was improved by 1.5–2-fold compared prior KPs.

The steady-state bidirectional performance of the 3D-KPs can be compared to prior work in terms of the normalized flow rate, blocking pressure, and power consumption. Considering that input power contributes to both flow rate and blocking pressure, an appropriate comparison metric of the bidirectional performance (*η*) is:

(4) $$\eta =\frac{\bar{Q_{ua}}\cdot \bar{\Delta P_{eq}}}{{\bar{W_{p}}}^{2}}$$

where $\bar{Q_{ua}}$ is the average of the flow rate normalized by the pumping area in both directions; $\bar{\Delta P_{eq}}$ is the average of the blocking pressure in both pumping directions; $\bar{W_{p}}$ is the average of the input power applied in both pumping directions for the highest performance. The bidirectional performance of the 3D-KP.MCE is calculated to be 70.4 sccm/cm^2^·Pa/W^2^, showing 1.5–2-fold improvement over prior bidirectional KPs (Figure 14).

Additionally, relative to prior work, the number of components was reduced by integrating the heat sinks and the gas routing channels in the 3D-printed thermal management platforms. The thermal management platforms also offer mechanical protection to the other components. The architecture requires only a few manual assembly steps, which are simple and repeatable.

Whereas MCE and AAO membranes were both successfully used as the pumping media, the lower thermal conductivity of the MCE membranes offered more efficient performance. The smaller pore size also significantly diminishes the opposing viscous flow. In contrast, the higher temperature tolerance of AAO membranes could be helpful for applications in hotter ambient conditions. The test results showed that AAO membranes remain functional at temperatures up to ≈200 °C, while providing nearly twice the flow rate of MCE membranes.

The forward pumping and reverse pumping performances were almost symmetrical as a consequence of the symmetrical architecture. A minor discrepancy was caused by the different thermal conditions of the thermal management platform which was on the lower side of KP. The upper thermal management platform experienced more natural convection than the lower thermal management platform, which also had more restricted air flow because of the underlying support. To better match the forward and reverse pumping performances, both thermal management platforms could be oriented perpendicular to the support.

Overall, the 3D-KP architecture resulted in a bidirectional Knudsen pump with superior thermal management, low system complexity, and improved bidirectional performance. The customized design addressed the contradictory requirements of thermal management for bidirectional pumping. The superior thermal management contributed to concentrated heating on the hot side of the 3D-KP, with minimized waste of heat through the heat sinks. The heat dissipation on the cool side remained effective at the same time. The performance of the 3D-KP was demonstrated to be sufficient to support certain microscale gas chromatography applications.

A number of improvements can be envisioned for future designs of 3D-printed bidirectional KPs. For example, micro laser sintering technology could be utilized to enable a finer and lighter thermal management platform with a much reduced total thermal mass [29]. The reduction of the thermal mass would lead to faster heating and cooling, and consequently improve the transient performance. In addition, the response time would also be shortened by close-loop control of the heating [30]. Separately, in order to fully benefit from the temperature range of AAO membranes, the various components attached to the KP should also be able to endure high temperatures. Possible alternatives to the nylon components used in this work include commercial polytetrafluoroethylene (PTFE) and polyether ether ketone (PEEK) counterparts [31].

## Figures and Tables

**Table 1:** Major simulated parameters in COMSOL.

Thermal conductivity (W·m^−1^·K^−1^)			
AlSi_10_Mg	179	MCE membrane	0.1
Air	0.02	Polyimide heater	0.46
Specific heat capacity (J·kg^−1^·K^−1^)			
AlSi_10_Mg	739	MCE membrane	860
Air	1000	Polyimide heater	400
Ambient Temperature (°C)			25
Natural convection coefficient (W·m^−2^·K^−1^)			5

**Table 2:** Specifications of DMLS process and material properties.

DMLS specifications			
Min. layer resolution (μm)	20	Minimum feature size (mm)	0.38
Surface roughness (μm)	Ra 7–10; Rz 50–60		
Typical tolerance (mm)	≈0.1		
AlSi_10_Mg properties			
Thermal conductivity (W·m^−1^·K^−1^)	179	Density (kg/m^3^)	2680

**Table 3:** Measured temperatures for 3D-KPs.

Power Levels	Temperatures for Forward Pumping (°C)			Temperatures for Reverse Pumping (°C)		
	*T_hot_*	*T_cool_*	Δ*T*	*T_hot_*	*T_cool_*	Δ*T*
3D-KP.MCE						
1 W	45.8	30.4	15.4	45.3	31.7	13.6
2 W	73.6	35.9	37.7	74.9	38.4	36.5
3D-KP.AAO						
2 W	61.5	42.9	21.6	64.3	43.4	20.9
9.8 W	194.3	73.1	121.2	191.2	73.2	118
